# Dental management of oral adverse effects associated with immune checkpoint inhibitors: A review

**DOI:** 10.17305/bb.2026.14252

**Published:** 2026-05-12

**Authors:** Andra Piciu, Andrei Tent, Dacian Sabau, Alexandru Mester

**Affiliations:** 1 Department of Medical Oncology, University of Medicine and Pharmacy “Iuliu Hatieganu”, Cluj-Napoca, Romania; 2 Department of Oral and Maxillo-Facial Surgery, Faculty of Medicine and Pharmacy, University of Oradea, Oradea, Romania; 3 Department of Oral Health, University of Medicine and Pharmacy ”Iuliu Hatieganu”, Cluj-Napoca, Romania

**Keywords:** Immune checkpoint inhibitors, immune-related adverse events, oral manifestations, xerostomia, lichenoid reactions, dental management

## Abstract

Cancer immunotherapy, particularly immune checkpoint inhibitors (ICIs) that target programmed cell death protein 1 (PD-1), programmed death-ligand 1 (PD-L1), and cytotoxic T-lymphocyte–associated antigen 4 (CTLA-4), has significantly advanced the field of oncology. However, these therapies are associated with immune-related adverse events that can affect various tissues, including the oral cavity. This narrative review consolidates current evidence regarding the immunopathogenesis, clinical presentation, differential diagnosis, and dental management of immunotherapy-related oral adverse effects. Relevant literature was identified through structured searches of the PubMed/MEDLINE, Scopus, and Web of Science databases. Oral manifestations of immunotherapy include xerostomia resulting from salivary gland dysfunction, oral lichenoid reactions, mucositis-like lesions, dysgeusia, dysphagia, periodontal inflammation, and, less commonly, autoimmune blistering disorders. These immune-mediated conditions are heterogeneous and may arise unpredictably during or after treatment. Accurate differential diagnosis is crucial, as these oral findings can mimic other therapy-related or infectious conditions. Management primarily focuses on supportive care and immunomodulatory approaches, including topical corticosteroids, saliva substitutes, and antimicrobial therapy when necessary, with systemic treatment reserved for severe cases. Early recognition and interdisciplinary collaboration are vital to minimize complications and ensure continuity of oncologic treatment. Further studies are required to elucidate the underlying mechanisms and enhance management strategies.

## Introduction

Over the past decade, cancer immunotherapy has dramatically transformed the therapeutic landscape of oncology, leading to sustained responses and improved survival rates across various malignancies. Immune checkpoint inhibitors (ICIs), which target pivotal regulatory pathways such as programmed cell death protein 1 (PD-1), programmed death-ligand 1 (PD-L1), and cytotoxic T-lymphocyte–associated antigen 4 (CTLA-4), have emerged as foundational treatments for cancers including melanoma, non-small cell lung cancer, renal cell carcinoma, and head and neck malignancies [1–3]. By rejuvenating antitumor immune activity, these agents enhance T-cell responses against malignant cells; however, this immune activation is not tumor-specific and may also impact normal tissues.

Consequently, immunotherapy is linked to a distinct range of toxicities referred to as immune-related adverse events (irAEs), which can affect almost any organ system [4]. Unlike the direct cytotoxic effects seen with chemotherapy or the localized damage associated with radiotherapy, irAEs are primarily immune-mediated and often resemble autoimmune or inflammatory conditions. While dermatologic, gastrointestinal, endocrine, and pulmonary toxicities are among the most frequently documented, involvement of the oral cavity and orofacial region is increasingly acknowledged but remains relatively underexplored [5, 6].

Oral irAEs encompass a diverse array of conditions, including xerostomia, salivary gland dysfunction, oral lichenoid reactions, mucositis-like lesions, dysgeusia, and oropharyngeal pain or dysphagia [6–8]. These manifestations can significantly disrupt essential functions such as mastication, swallowing, and speech, adversely affecting nutritional status, quality of life, and overall well-being. Furthermore, oral complications may hinder adherence to cancer therapy, necessitate dose modifications, or lead to treatment interruptions in severe cases [9]. Despite their clinical significance, oral toxicities are frequently underdiagnosed or misattributed, partly due to overlapping features with other treatment-related effects and a lack of standardized diagnostic criteria.

From a dental perspective, the increasing application of immunotherapy poses new challenges in patient management. Dentists often serve as the first clinicians to identify oral abnormalities and play a vital role in early detection, differential diagnosis, and supportive care. However, awareness of immunotherapy-related oral adverse effects is limited in dental practice, and evidence-based guidelines for their management are still evolving [6–10].

A structured literature search was conducted to identify pertinent publications on ICI-related oral adverse effects and their dental management. Electronic databases, including PubMed, Scopus, and Web of Science, were searched for articles published up to 2025 using combinations of keywords such as “immune checkpoint inhibitors,” “oral adverse events,” “immune-related adverse events,” “xerostomia,” “oral lichenoid reactions,” and “dental management.” The review included clinical studies, observational studies, narrative and systematic reviews, and relevant clinical practice guidelines. Articles were selected based on relevance to the topic, recency of publication, and clinical applicability. Studies that did not address ICIs or were unrelated to oral manifestations were excluded. Emphasis was placed on widely cited studies and international guidelines to ensure a clinically meaningful synthesis of current evidence.

While several recent reviews have examined the pathophysiology and clinical spectrum of oral irAEs, their implications for dental practice remain insufficiently structured. This review aims to provide a clinically oriented synthesis focused on dental management, including a practical classification framework, diagnostic approach, and management strategies tailored to dental practitioners involved in the care of patients receiving ICIs.

**Figure 1.**
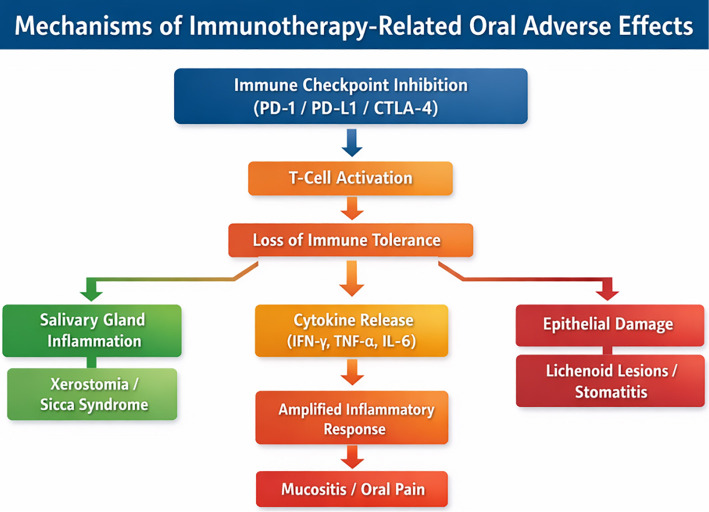
**Proposed mechanisms underlying immunotherapy-related oral adverse events.** Immune checkpoint inhibitors targeting PD-1, PD-L1, and CTLA-4 enhance T-cell activation and disrupt peripheral immune tolerance, thereby promoting immune-mediated injury in oral tissues. Salivary gland inflammation may lead to xerostomia and sicca syndrome, whereas cytokine release, including IFN-γ, TNF-α, and IL-6, can amplify local inflammatory responses and contribute to mucositis and oral pain. In parallel, epithelial damage may result in lichenoid lesions and stomatitis. Together, these pathways illustrate how checkpoint blockade may induce heterogeneous oral immune-related adverse events involving both salivary gland dysfunction and mucosal injury. Abbreviations: CTLA-4, cytotoxic T-lymphocyte–associated antigen 4; ICIs, immune checkpoint inhibitors; IFN-γ, interferon gamma; IL-6, interleukin 6; irAEs, immune-related adverse events; PD-1, programmed cell death protein 1; PD-L1, programmed death-ligand 1; TNF-α, tumor necrosis factor alpha.

## Overview of ICIs relevant to oral toxicity

Cancer immunotherapy encompasses a variety of therapeutic strategies designed to enhance the host immune response against malignant cells. Among these strategies, ICIs represent the most widely utilized class in current clinical practice [11, 12]. These agents target inhibitory signaling pathways that regulate T-cell activation, primarily involving PD-1, its ligand PD-L1, and CTLA-4 [11, 12]. Monoclonal antibodies directed against these checkpoints—such as anti–PD-1, anti-PD-L1, and anti-CTLA-4 therapies—have demonstrated significant efficacy across a broad spectrum of solid tumors and hematologic malignancies [11, 12].

Under physiological conditions, immune checkpoints play a critical role in maintaining self-tolerance and preventing excessive immune activation. Tumor cells exploit these pathways by upregulating ligands such as PD-L1, thereby inhibiting cytotoxic T-cell responses and evading immune surveillance. ICIs restore antitumor immunity by blocking these inhibitory interactions, leading to sustained activation and proliferation of effector T cells [13].

However, this non-specific immune activation also disrupts peripheral tolerance and may result in immune-mediated damage to normal tissues. Consequently, ICIs are associated with a unique spectrum of irAEs, which can affect multiple organ systems, including the skin, gastrointestinal tract, endocrine glands, lungs, and, less commonly, the oral cavity [4, 9]. The pathogenesis of irAEs is complex and involves autoreactive T-cell activation, increased cytokine production, and, in some cases, autoantibody formation, collectively resembling autoimmune or inflammatory conditions [14–16].

In the oral and maxillofacial region, these immune-mediated effects may target the mucosal epithelium and salivary glands, leading to clinical manifestations such as xerostomia, mucosal inflammation, and lichenoid lesions. Importantly, these toxicities differ mechanistically and clinically from those induced by conventional chemotherapy or radiotherapy, as they are not dose-dependent and may occur at variable time points during or even after cessation of immunotherapy [7, 9, 16]. For dental practitioners, understanding these mechanisms is essential for recognizing atypical presentations and distinguishing immunotherapy-related lesions from other treatment-related or infectious conditions [7, 9, 17].

## Pathophysiology of oral irAEs

The pathophysiology of oral irAEs is not yet fully understood, but current evidence suggests that these complications arise from dysregulated immune activation following checkpoint inhibition [18, 19] (Figure 1). By blocking inhibitory pathways such as PD-1/PD-L1 and CTLA-4, ICIs disrupt peripheral tolerance mechanisms, leading to enhanced T-cell activation not only against tumor cells but also against normal tissues [18, 19].

A central mechanism involves the expansion of autoreactive T-cell clones and increased production of pro-inflammatory cytokines, including interferon-γ and tumor necrosis factor-α, which contribute to epithelial damage and chronic mucosal inflammation [20]. In the oral cavity, this immune dysregulation can target both the stratified squamous epithelium and the minor and major salivary glands, resulting in a spectrum of mucosal and glandular alterations [9, 20].

Salivary gland involvement is a prominent feature of oral irAEs and often clinically manifests as xerostomia or sicca syndrome. Histopathological studies have demonstrated focal lymphocytic sialadenitis with a predominance of CD8+ T cells, distinguishing immunotherapy-related sicca from classical Sjögren syndrome, which is typically characterized by CD4+ T-cell and B-cell infiltration and autoantibody production [21, 22]. Additionally, immunotherapy-induced salivary dysfunction may present abruptly and with more severe glandular hypofunction compared to primary autoimmune conditions [21, 22].

Mucosal lesions, particularly lichenoid reactions, are thought to result from cytotoxic T-cell-mediated apoptosis of basal keratinocytes, a mechanism similar to that observed in oral lichen planus. However, immunotherapy-related lichenoid lesions may exhibit distinct clinical and histological features, including a more intense inflammatory infiltrate and variable distribution, suggesting a drug-induced immune-mediated process [23]. Increased antigen presentation and loss of tolerance to epithelial antigens are believed to play a key role in this context [23].

Furthermore, emerging evidence indicates that humoral immunity may also contribute to certain irAEs, particularly in cases resembling autoimmune blistering diseases such as pemphigoid-like or pemphigus-like lesions, where autoantibody production has been reported [24]. The interplay between cellular and humoral immune responses likely underlies the heterogeneity of oral manifestations.

Importantly, unlike chemotherapy- or radiotherapy-induced oral toxicities, which are primarily dose-dependent and directly cytotoxic, immunotherapy-related oral adverse effects are immune-mediated, unpredictable, and may occur at any stage of treatment or even after therapy discontinuation [4, 25]. This variability in onset and presentation poses diagnostic challenges and underscores the importance of clinician awareness.

Recent advances in understanding immune checkpoint inhibition have further elucidated the complexity of irAEs [26]. A conceptual analysis by Wraith and Nicholson underscores that checkpoint blockade not only enhances antitumor immunity but also disrupts essential mechanisms of immune homeostasis, including peripheral tolerance, regulatory T-cell function, and immune network balance [26]. This comprehensive perspective suggests that irAEs may result from systemic immune recalibration rather than isolated autoreactive processes, involving both innate and adaptive immune components. Such insights may clarify the heterogeneity, unpredictability, and organ-specific manifestations of irAEs, including those affecting the oral cavity, and highlight the necessity for further mechanistic studies to refine diagnostic and therapeutic strategies [26, 27].

For dental practitioners, understanding these underlying mechanisms is crucial for accurate diagnosis and guiding management strategies, as many oral irAEs respond to immunomodulatory therapies rather than conventional symptomatic treatments alone. However, the relative contributions of these mechanisms may vary across different oral manifestations, and the underlying pathophysiology for several clinical entities remains incompletely defined [4, 9, 21–23].

## Spectrum of immunotherapy-related oral adverse effects

Current evidence categorizes oral irAEs into well-established entities with consistent clinical and histopathological characteristics and less well-defined or emerging associations, for which data remain limited or heterogeneous [7, 28]. Their clinical presentation varies widely, ranging from mild, self-limited symptoms to severe, functionally debilitating conditions. Although reported prevalence differs across studies, these manifestations are increasingly recognized in patients receiving ICIs and may occur at any stage of treatment [4, 7] (Table 1). In this review, oral conditions in patients receiving ICIs are classified into: primary oral irAEs; secondary complications associated with irAEs; and non-irAE conditions included in the differential diagnosis.

**Table 1:** Oral adverse effects associated with immunotherapy

**Adverse effects**	**Clinical presentation**	**Typical onset**	**Severity**	**Frequency/Evidence level**	**Key diagnostic clues**	**Ref**
Primary oral immune-related adverse events (irAEs**)**						
Xerostomia/sicca syndrome	Oral dryness, dysphagia, altered taste, difficulty speaking; increased caries risk	Typically, weeks to months after initiation	Mild to severe; may be persistent	Common (reported ∼5–30%); well-established	Reduced salivary flow, mucosal dryness, rapid onset compared to radiation; CD8+ predominance (histology)	[6, 7, 15–17]
Oral lichenoid reactions	Reticular white striae, erythema, erosions, ulcerations; often bilateral	Typically weeks to months after initiation	Mild to severe; may be painful (erosive forms)	Relatively common (∼1–10%); well-established	OLP-like lesions but atypical distribution; temporal link with ICI therapy	[17, 18]
Stomatitis/mucositis-like lesions	Diffuse erythema, ulcerations, burning sensation, oral pain	Unpredictable; not treatment-cycle dependent	Moderate to severe; may impair oral function	Moderately reported (∼5–15%); well-established	Unlike chemotherapy mucositis: irregular onset, prolonged course, immune-mediated	[9, 17, 21, 22]
Dysgeusia	Altered or reduced taste, metallic taste, anorexia	Variable; may occur early or late	Mild to moderate; functional impact variable	Variable frequency (∼2–10%); limited data	Often underreported; associated with salivary changes or mucosal inflammation	[9, 10]
Dysphagia/oropharyngeal symptoms	Difficulty swallowing, throat pain, sensation of obstruction	Variable; may occur at any stage	Mild to severe; may affect oral intake	Less common (<5%); limited data	Often associated with xerostomia or mucosal lesions; may require multidisciplinary evaluation	[10, 19]
Periodontal/gingival inflammation	Gingival erythema, bleeding, exacerbation of periodontitis	Gradual or coinciding with therapy	Mild to moderate; potentially progressive	Emerging; incidence not well defined	Disproportionate inflammation vs plaque; immune dysregulation suspected	[20]
Autoimmune blistering-like lesions	Bullae, erosions, desquamative gingivitis	Variable	Moderate to severe	Rare (<1%); well-described	Pemphigoid/pemphigus-like features; requires biopsy/immunofluorescence	[14, 18]
Severe mucocutaneous reactions (e.g., Stevens–Johnson syndrome)	Extensive ulceration, mucosal sloughing, severe pain	Acute	Severe; potentially life-threatening	Very rare (<0.1%); severe	Rapid progression, systemic involvement, requires urgent care	[14, 18]
**Secondary complications associated with oral irAEs**						
Oral candidiasis	White plaques, erythema, burning sensation	Secondary to mucosal damage or xerostomia	Mild to moderate	Secondary complication (incidence variable, up to ∼10–20% in high-risk patients); context-dependent	Response to antifungal therapy; microbiological confirmation. May also mimic immune-related mucosal lesions; requires differentiation	[21, 22]

Frequency, incidence, and evidence levels are determined based on the existing literature and may vary across studies due to heterogeneity in reporting. Additionally, secondary complications may overlap with conditions included in the differential diagnosis. Abbreviations: ICI, immune checkpoint inhibitor; irAEs, immune-related adverse events; OLP, oral lichen planus.

### Xerostomia and Sicca syndrome

Xerostomia is one of the most frequently reported and well-established oral irAEs, with variable incidence across studies and typical onset occurring within weeks to months after therapy initiation. Patients typically present with oral dryness, difficulty in mastication and swallowing, altered taste, and increased fluid intake. Clinical examination may reveal reduced salivary pooling, mucosal dryness, and increased susceptibility to dental caries, candidiasis, and periodontal disease [7, 27, 28].

Unlike radiation-induced salivary gland damage, which is dose-dependent and often irreversible, immunotherapy-related sicca syndrome may have a more variable course. Histologically, it is characterized by lymphocytic infiltration with a predominance of CD8+ T cells, distinguishing it from Sjögren syndrome [21, 22]. Early recognition is crucial, as persistent hyposalivation has significant implications for long-term oral health.

### Oral lichenoid reactions

Oral lichenoid reactions represent another well-characterized manifestation of ICI therapy, typically occurring weeks to months after treatment initiation and varying in severity from mild reticular lesions to painful erosive forms. Clinically, they may present as reticular white striations, erythematous or erosive lesions, and, in some cases, painful ulcerations. These lesions often resemble oral lichen planus but may exhibit atypical distribution or severity [23, 29]. Symptoms range from mild discomfort to severe pain that interferes with oral intake. Histopathological features typically include a band-like lymphocytic infiltrate and basal cell degeneration, consistent with a T-cell–mediated immune response. Recognition of these lesions is important, as they may require topical or systemic immunosuppressive therapy depending on severity [23, 29].

### Stomatitis and mucositis-like lesions

Stomatitis associated with immunotherapy may present as diffuse erythema, ulcerations, or nonspecific inflammatory lesions of the oral mucosa. Although these lesions may resemble chemotherapy-induced mucositis, their pathogenesis is immune-mediated rather than directly cytotoxic [9, 28]. Clinically, patients may report burning sensations, pain, and difficulty eating. Lesions can involve any oral site and vary in severity. Unlike classical mucositis, the timing of onset is less predictable, and lesions may persist or recur despite standard supportive care [10, 28, 29].

### Dysgeusia

Dysgeusia is a relatively common but undercharacterized oral manifestation, with variable reported incidence and often mild to moderate severity, although it may significantly affect quality of life [6, 9]. Patients may experience reduced taste sensitivity, a metallic taste, or complete taste loss. Dysgeusia can substantially impact appetite, nutritional intake, and overall quality of life [9, 10]. The underlying mechanisms are not fully understood but may involve inflammatory changes in taste receptors, salivary alterations, or neural involvement. Given its subtle presentation, dysgeusia may be overlooked unless specifically assessed during clinical evaluation [9, 10].

### Dysphagia and oropharyngeal symptoms

Dysphagia and oropharyngeal discomfort may occur in association with mucosal inflammation, xerostomia, or neuromuscular involvement. Patients may report difficulty swallowing, throat pain, or a sensation of food sticking [10, 27]. These symptoms are clinically significant, as they may lead to dehydration, malnutrition, and reduced adherence to cancer therapy. In some cases, multidisciplinary evaluation is required to exclude esophageal or neurological involvement [10, 27].

### Periodontal and gingival manifestations

Periodontal and gingival manifestations are emerging associations of ICI therapy, with limited and heterogeneous evidence regarding their incidence, timing, and underlying mechanisms. Reported findings include gingival erythema, bleeding, and exacerbation of pre-existing periodontal disease [20, 25]. Although the exact mechanisms remain unclear, immune dysregulation and altered host–microbiome interactions are thought to contribute [25]. This area remains under-investigated but is particularly relevant for dental practitioners, given its potential impact on periodontal stability and long-term oral health [20].

### Severe oral manifestations

Less common but clinically significant oral irAEs include autoimmune blistering diseases such as mucous membrane pemphigoid–like and pemphigus-like lesions, as well as severe mucocutaneous reactions resembling Stevens–Johnson syndrome or toxic epidermal necrolysis [24, 29]. These conditions may present with extensive ulceration, bullae, or desquamative lesions and are often associated with significant pain and functional impairment. Prompt recognition and referral are critical, as these entities may require systemic immunosuppressive therapy and can be potentially life-threatening [24, 29].

Oral candidiasis may play a dual role in this context: it can occur as a secondary complication of oral irAEs (e.g., due to xerostomia or mucosal disruption), but it may also represent a differential diagnosis, as its clinical presentation can mimic immune-mediated mucosal lesions [24, 29].

## Differential diagnosis

This section focuses on conditions that may mimic oral irAEs and must be distinguished from both primary immune-mediated lesions and secondary complications. The diagnosis of irAEs can be challenging due to their clinical overlap with a wide range of oral conditions commonly encountered in oncology patients [20, 24, 29]. Accurate differentiation is essential to ensure appropriate management, avoid unnecessary interruption of cancer therapy, and prevent misdiagnosis of potentially serious conditions. A structured approach based on clinical history, timing of symptom onset, lesion characteristics, and adjunctive investigations is recommended [20, 24, 29] (Table 2). The distinction between well-established oral irAEs and less clearly defined or emerging manifestations is particularly important, as it may influence diagnostic confidence and guide appropriate management strategies.

**Table 2:** Conditions not related to irAEs in the differential diagnosis of oral lesions

**Condition**	**Clinical features**	**Key differentiating features from oral irAEs**	**Diagnostic approach**	**Frequency/Clinical relevance**	**Ref**
Chemotherapy-induced mucositis	Diffuse erythema, ulcerations, severe pain, often widespread	Predictable onset (5–10 days post-chemotherapy); cycle-dependent; resolves between cycles	Clinical history, timing relative to chemotherapy cycles	Common in oncology patients; well-established	[21, 22]
Radiation-induced xerostomia / mucosal injury	Persistent dry mouth, mucosal atrophy, fibrosis, increased caries risk	Dose-dependent; irreversible salivary gland damage; confined to radiation field	Radiation history (dose, field), salivary flow assessment	Common in head and neck radiotherapy; well-established	[10, 16]
Oral candidiasis	White plaques (pseudomembranous), erythematous lesions, burning sensation	Lesions are often scrapable; responds to antifungals; may be secondary to xerostomia. May mimic oral irAEs; however, typically responsive to antifungal therapy and often associated with predisposing factors such as xerostomia	Clinical exam, microbiological testing, therapeutic response	Common secondary infection; well-established	[21, 22]
Herpes simplex virus (HSV) infection	Painful clustered ulcers, often on keratinized mucosa; may recur	Acute onset; vesicle-to-ulcer evolution; responsive to antivirals	PCR, viral culture, clinical pattern	Common opportunistic infection; well-established	[24]
Pre-existing autoimmune diseases (e.g., oral lichen planus, Sjögren syndrome)	Chronic mucosal lesions, xerostomia, possible systemic features	History of autoimmune disease; gradual onset; systemic involvement possible	Biopsy, immunological tests (autoantibodies), clinical history	Less common but clinically important	[25]
Drug-induced oral reactions (non-ICI)	Lichenoid lesions, ulcerations, xerostomia	Temporal association with specific drugs; resolution after withdrawal	Medication review, dechallenge/rechallenge (if feasible)	Variable; requires careful medication review	[26]
Tumor-related lesions / disease progression	Non-healing ulcers, induration, mass lesions, bleeding	Persistent, progressive lesions; lack of response to symptomatic therapy	Biopsy, imaging, oncologic evaluation	Critical to exclude; high clinical significance	[27]
Bacterial infections / periodontal disease	Gingival inflammation, bleeding, periodontal pockets, halitosis	Correlates with plaque accumulation; localized periodontal findings; not immune-mediated	Periodontal examination, probing depth, radiographs	Depends on oral hygiene and baseline status	[20, 25]

Abbreviations: HSV, herpes simplex virus; ICI, immune checkpoint inhibitor; irAEs, immune-related adverse events; PCR, polymerase chain reaction.

### Chemotherapy-induced oral lesions

Chemotherapy-induced oral mucositis is a prevalent complication among cancer patients and can closely resemble immunotherapy-related stomatitis. Its pathogenesis primarily involves direct cytotoxic effects on rapidly dividing epithelial cells, resulting in mucosal atrophy, ulceration, and subsequent inflammation [19, 20]. Clinically, chemotherapy-induced mucositis typically follows a predictable temporal pattern, with onset occurring within 5–10 days after drug administration and resolution within 2–3 weeks, depending on the specific regimen [22]. In contrast, irAEs in the oral cavity are immune-mediated, exhibit less predictable onset, and may persist or recur despite standard supportive care. It is crucial to distinguish between these conditions, as immunotherapy-related lesions may respond more favorably to immunomodulatory therapies [20–22].

### Radiation-induced oral changes

Radiotherapy targeting the head and neck region is associated with a well-defined spectrum of oral complications, which includes mucositis, xerostomia, fibrosis, and an increased susceptibility to infections and dental caries [10]. Radiation-induced xerostomia results from irreversible damage to salivary gland acinar cells and is typically dose-dependent. This differs from immunotherapy-related salivary dysfunction, which is immune-mediated and may vary in severity, with some cases exhibiting partial reversibility [16]. A thorough oncologic history, including details of radiation fields and cumulative doses, is essential for accurate differentiation [16].

### Infectious conditions

Opportunistic infections are prevalent in immunocompromised cancer patients and must be systematically ruled out. Oral candidiasis can manifest as pseudomembranous plaques, erythematous lesions, or angular cheilitis [21]. In oncology patients, particularly those experiencing salivary dysfunction or mucosal damage, candidiasis is a common opportunistic infection [22]. It also serves as an important differential diagnosis in patients undergoing immunotherapy, as clinical features may overlap with immune-related mucosal lesions [5, 6]. Consequently, candidiasis should be considered both a potential complication of irAEs and a significant diagnostic alternative.

Unlike candidiasis, herpes simplex virus (HSV) infection represents a distinct differential diagnosis. HSV can cause painful ulcerations that may mimic mucositis or immune-mediated lesions [29]. In the context of immunotherapy, distinguishing between infection and irAEs is particularly critical, as inappropriate use of corticosteroids in the presence of undiagnosed infections may exacerbate the condition. Microbiological testing, response to antifungal or antiviral therapy, and clinical evolution are instrumental in establishing the diagnosis [22, 24].

### Pre-existing autoimmune diseases

Autoimmune disorders affecting the oral cavity, such as oral lichen planus and Sjögren’s syndrome, may exhibit clinical features similar to those of immunotherapy-related lesions. In some instances, ICIs may exacerbate previously undiagnosed or subclinical autoimmune diseases [25]. A comprehensive medical history is vital for identifying any prior autoimmune diagnoses or symptoms indicative of underlying conditions. Histopathological examination and serological testing can aid in differentiating primary autoimmune disorders from drug-induced immune phenomena [6].

### Drug reactions unrelated to immunotherapy

Numerous medications commonly administered to oncology patients, including antibiotics, antifungals, and supportive care drugs, may induce oral adverse reactions such as lichenoid drug eruptions, mucosal ulcerations, or xerostomia [26]. Drug-induced oral lesions frequently exhibit a temporal relationship with medication initiation and may resolve upon discontinuation of the offending agent. Differentiating these reactions from immunotherapy-related effects necessitates a careful review of the patient’s medication history and, in some cases, a trial of drug withdrawal [26].

### Tumor-related lesions and disease progression

Oral lesions in cancer patients may signify primary tumors, metastatic disease, or local extension of malignancy. Ulcerative, indurated, or non-healing lesions should raise suspicion for neoplastic involvement, particularly in patients with head and neck cancers [27]. Biopsy and histopathological evaluation are essential in cases of diagnostic uncertainty or when lesions fail to respond to conventional management. Misinterpreting tumor-related lesions as treatment-related toxicity may delay oncologic diagnosis and negatively impact patient outcomes [27].

### Implications for dental management

The increasing utilization of ICIs in oncology necessitates that dental practitioners become proficient in recognizing and managing irAEs [10, 19, 20]. Given their potential impact on oral function, quality of life, and the continuation of cancer therapy, a proactive and structured dental approach is essential. Management strategies should be tailored to the type and severity of oral manifestations, with close collaboration between dental and oncology teams [10, 19, 20] (Table 3). Unlike previous reviews that predominantly focus on medical management, this section emphasizes practical considerations pertinent to dental practitioners, including preventive care, procedural decision-making, and coordination with oncology teams.

**Figure 2.**
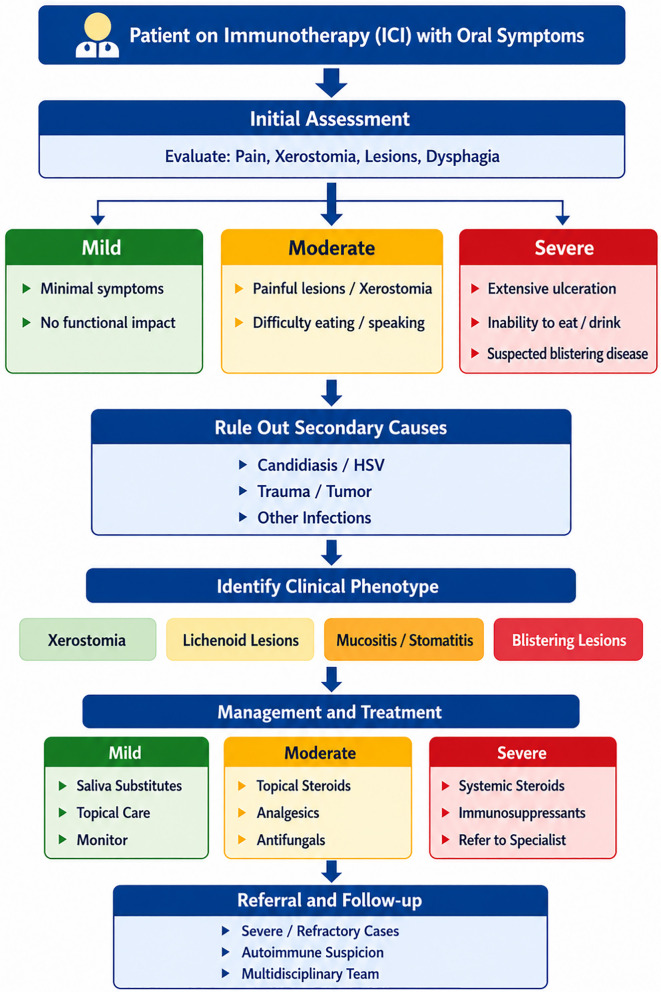
**Proposed clinical algorithm for the assessment and management of immunotherapy-related oral adverse events.** This conceptual algorithm outlines a stepwise approach for patients receiving ICI therapy who present with oral symptoms. Initial assessment should document pain, xerostomia, mucosal lesions, dysphagia, and functional impairment, followed by severity stratification into mild, moderate, or severe presentations. Secondary causes, including candidiasis, HSV infection, trauma, tumor-related lesions, and other infections, should be excluded before defining the dominant clinical phenotype. The algorithm focuses on the most common and clinically relevant mucosal and salivary manifestations, including xerostomia, lichenoid lesions, mucositis-like/stomatitis lesions, and autoimmune blistering disorders. Management is guided by severity and may range from saliva substitutes, topical care, monitoring, analgesics, topical corticosteroids, and antifungal therapy to systemic corticosteroids, immunosuppressive treatment, specialist referral, and multidisciplinary follow-up in severe or refractory cases. The algorithm is adapted from available literature and clinical practice recommendations [4, 10, 28, 31] and has not been prospectively validated. It does not encompass the full spectrum of ICI-associated oral adverse effects, such as dysgeusia, isolated oropharyngeal symptoms, periodontal or gingival manifestations, and secondary infections, which require individualized evaluation and are addressed separately in the text and tables. Abbreviations: HSV, herpes simplex virus; ICI, immune checkpoint inhibitor.

**Table 3:** Recommended strategies for managing oral adverse effects related to immunotherapy [4, 6, 7, 9, 10, 28].

**Condition**	**Commonly used approaches**	**Additional considerations**	**Dental precautions**	**Indications for referral**
Xerostomia/sicca syndrome	Saliva substitutes, frequent hydration	Sugar-free chewing gum, sialagogues (pilocarpine/cevimeline if indicated), topical fluoride, antifungal prophylaxis, when indicated	High caries risk → intensive preventive care; avoid alcohol-containing rinses; monitor prosthesis tolerance	Severe hyposalivation, suspected autoimmune involvement, or refractory symptoms
Oral lichenoid reactions	Topical corticosteroids (e.g., clobetasol, dexamethasone rinse)	Topical anesthetics, antifungals (if secondary infection), calcineurin inhibitors (selected cases)	Avoid trauma (sharp teeth, ill-fitting prostheses); postpone elective procedures in erosive lesions	Extensive, erosive, or refractory lesions; diagnostic uncertainty (biopsy required)
Stomatitis/mucositis-like lesions	Topical corticosteroids, non-irritating oral care	Topical anesthetics, coating agents, antifungals if superinfection suspected	Delay invasive procedures in moderate–severe cases; maintain gentle oral hygiene	Severe pain, inability to eat/drink, or extensive ulcerations
Dysgeusia	Dietary counseling, oral hygiene optimization	Zinc supplementation (selected cases), saliva substitutes	Monitor nutritional intake; reinforce oral hygiene	Persistent symptoms significantly affecting nutrition or quality of life
Dysphagia/oropharyngeal symptoms	Symptomatic management, hydration	Nutritional support, speech/swallowing therapy	Avoid complex dental procedures if swallowing is impaired	Moderate–severe dysphagia, dehydration, or suspected systemic involvement
Periodontal/gingival inflammation	Professional cleaning, improved oral hygiene	Antimicrobial mouthrinses (e.g., chlorhexidine), local periodontal therapy	Close periodontal monitoring; increased recall frequency	Rapid progression or poor response to conventional therapy
Secondary infections	Antifungal or antiviral therapy (based on diagnosis)	Saliva substitutes, pain control	Avoid corticosteroids until infection is excluded; reinforce hygiene	Refractory infections or diagnostic uncertainty
Autoimmune blistering-like lesions (pemphigoid/pemphigus-like)	Topical corticosteroids (mild cases)	Systemic corticosteroids or immunosuppressants (specialist-guided)	Avoid trauma; defer elective procedures; soft diet	Urgent referral (oral medicine/dermatology); biopsy required
Severe mucocutaneous reactions (e.g., Stevens–Johnson syndrome)	Emergency supportive care	Systemic therapy in hospital setting	No dental procedures; supportive oral care only	Immediate hospital referral (medical emergency)

### Pre-treatment dental assessment

These recommendations are derived from general principles of oncology dental care, as specific data regarding immunotherapy are limited. Ideally, patients slated to receive immunotherapy should undergo a comprehensive dental evaluation prior to or early in the treatment process. The primary objectives are to identify and eliminate potential sources of oral infection and to establish a baseline for future comparisons [10, 19, 20]. This assessment may encompass evaluation of dental caries and existing restorations; periodontal examination; assessment of oral mucosa; baseline salivary function and xerostomia symptoms; and radiographic evaluation [5, 10, 28]. Preventive measures such as professional cleaning, treatment of active infections, and patient education on oral hygiene should be implemented. Although ICIs are not typically associated with the same level of immunosuppression as chemotherapy, maintaining optimal oral health reduces the risk of complications and facilitates early detection of irAEs [5, 10, 28].

### Monitoring during immunotherapy

Evidence supporting optimal monitoring frequency remains limited. Regular oral examinations are generally recommended throughout the course of immunotherapy, particularly for patients reporting symptoms such as dryness, pain, or taste alterations. Dentists should maintain a high level of suspicion for oral irAEs, especially given their variable onset and presentation [7]. Monitoring should include documentation of mucosal changes (location, size, severity); assessment of salivary flow and xerostomia symptoms; evaluation of pain and functional impairment (e.g., eating, speaking); and photographic documentation [7]. Early identification of oral toxicities enables timely intervention and may prevent progression to more severe forms requiring systemic therapy or interruption of oncologic treatment [7].

### Management of specific oral toxicities

Most management strategies are based on experience with autoimmune and other cancer therapy-related conditions rather than prospective trials in this specific context. The treatment of oral irAEs is primarily symptom-driven and depends on the underlying clinical presentation [6, 20, 28].

Therapy for xerostomia and salivary gland dysfunction in patients receiving immunotherapy is predominantly supportive, aimed at improving oral lubrication and preventing secondary complications. Patients should be encouraged to maintain adequate hydration and may benefit from saliva substitutes to alleviate symptoms of oral dryness [6, 7]. Mechanical stimulation of salivary flow, such as the use of sugar-free chewing gum, or pharmacologic sialagogues, when not contraindicated, can further enhance residual gland function [6, 10]. Preventive strategies, including the application of topical fluoride, are crucial for mitigating the increased risk of dental caries associated with hyposalivation [10, 28]. Additionally, antifungal prophylaxis or targeted antifungal therapy may be necessary for patients exhibiting clinical or microbiological evidence of candidiasis, particularly in the presence of persistent salivary dysfunction [6, 9].

Mucosal lesions, including lichenoid reactions and stomatitis, are typically managed with a combination of anti-inflammatory and supportive measures. Topical corticosteroids serve as the first-line therapy for mild to moderate lesions, effectively reducing immune-mediated inflammation and promoting mucosal healing [6, 20, 28]. Symptomatic relief can be achieved through topical anesthetics, especially in patients experiencing significant pain that interferes with oral intake [10]. Maintaining meticulous oral hygiene with non-irritating products is essential for preventing secondary complications and supporting mucosal recovery. Patients should also be advised to avoid irritative factors, such as spicy, acidic, or abrasive foods, which may exacerbate mucosal inflammation and impede healing [6, 10].

Secondary infections, including fungal and viral infections, should be promptly identified and managed appropriately. Antifungal or antiviral therapy should be initiated based on clinical presentation and, when necessary, confirmed by microbiological testing [24]. In cases of diagnostic uncertainty or when lesions fail to respond to initial therapy, further diagnostic evaluation, including laboratory or microbiological investigations, is recommended to guide targeted treatment [22].

These approaches align with current clinical practice recommendations, which emphasize topical immunosuppressive therapy and supportive care as first-line interventions for many oral irAEs [6].

### Dental procedures in patients receiving immunotherapy

Data regarding procedural safety in patients undergoing immunotherapy are limited. Generally, routine dental care can be safely performed in this population, provided that oral toxicities are mild or well-controlled. However, caution is warranted in the presence of active mucosal lesions, severe xerostomia, or significant pain [6, 7, 9]. Key considerations include postponing elective invasive procedures in patients with severe oral irAEs, minimizing trauma to fragile mucosa, ensuring adequate pain control, and closely monitoring healing following surgical procedures [6, 7, 9]. Unlike patients receiving chemotherapy, neutropenia and thrombocytopenia are less common with ICIs; however, immune-mediated complications may still affect healing and patient tolerance [7, 9].

### Indications for referral and multidisciplinary care

Referral criteria are primarily based on expert opinion and clinical practice patterns. Prompt referral and interdisciplinary collaboration are essential in managing moderate to severe oral irAEs (Figure 2, Table 3). Figure 2 presents a simplified, clinically oriented algorithm focused on major mucosal and salivary manifestations, while other presentations (e.g., dysgeusia, dysphagia, periodontal changes, and secondary infections) are addressed separately in the text and tables. Referral to oncology and/or oral medicine specialists is indicated in cases of severe or persistent mucosal ulcerations, extensive lichenoid or erosive lesions, significant dysphagia or inability to maintain oral intake, suspected autoimmune blistering diseases, or lack of response to first-line topical therapy [4–6]. Management of these patients may require systemic corticosteroids or other immunosuppressive agents, which must be coordinated with the oncology team to balance toxicity control with oncologic efficacy [4–6].

## Multidisciplinary approach

The management of irAEs necessitates a coordinated multidisciplinary approach involving oncologists, dental practitioners, oral medicine specialists, and other healthcare professionals. Given the complex and immune-mediated nature of these toxicities, effective communication between disciplines is crucial to ensure accurate diagnosis, timely intervention, and optimal patient outcomes. This perspective highlights the unique role of dental practitioners, which is often underrepresented in the existing literature on immunotherapy-related toxicities [6, 28, 29].

Oncologists play a central role in initiating and monitoring immunotherapy, as well as assessing the severity of systemic immune-related toxicities and determining the need for treatment modification or interruption. However, oral manifestations are frequently first identified by dental professionals, who are uniquely positioned to detect early mucosal and salivary changes during routine examinations. Early recognition by dentists can facilitate prompt management and prevent progression to more severe complications [6, 7].

Oral medicine specialists contribute to the differential diagnosis and management of complex cases, particularly when lesions are atypical, persistent, or resistant to first-line therapy. Histopathological evaluation, immunofluorescence studies, and targeted investigations may be necessary to distinguish oral irAEs from autoimmune diseases, infections, or neoplastic processes. In cases of severe mucosal involvement or suspected autoimmune blistering disorders, collaboration with dermatologists is often required [31].

Additionally, patients with significant xerostomia, dysphagia, or nutritional compromise may benefit from input from nutritionists and speech and swallowing specialists. These professionals are essential in maintaining adequate nutritional intake, preventing weight loss, and improving functional outcomes. In selected cases, rheumatologists or immunologists may be involved in managing systemic immune-mediated conditions associated with immunotherapy [9].

A structured communication pathway between dental and oncology teams is critical. Dentists should promptly report significant oral findings, particularly those that may impact the patient’s ability to continue cancer therapy. Conversely, oncologists should inform dental providers about the type of immunotherapy, treatment timeline, and any history of irAEs. This bidirectional exchange of information supports individualized patient care and minimizes the risk of misdiagnosis or delayed intervention [6, 20, 28].

Current clinical practice statements emphasize that integrating oral healthcare into oncology care pathways improves both oral and systemic outcomes. Early dental involvement, preventive strategies, and ongoing monitoring should be considered standard components of care for patients receiving immunotherapy [6, 10].

## Gaps in knowledge and future directions

Despite the growing recognition of oral irAEs, the current evidence base remains limited and characterized by significant methodological and clinical heterogeneity [6, 20, 28]. Addressing these limitations is essential to improve patient outcomes and support the development of evidence-based clinical guidelines.

A major challenge is the lack of standardized definitions and diagnostic criteria for oral irAEs. Current data are largely derived from case reports, retrospective studies, and heterogeneous cohorts, resulting in wide variability in reported prevalence and clinical presentation [7, 8]. The absence of validated grading systems specifically tailored to oral toxicities further complicates the comparison of outcomes across studies and limits the establishment of clear management algorithms.

Another important gap concerns the pathophysiological mechanisms underlying oral irAEs. While T-cell- mediated immune dysregulation is widely accepted as a central process, the specific molecular pathways involved in different oral manifestations, such as salivary gland dysfunction vs mucosal inflammation, remain incompletely defined. The role of the oral microbiome, genetic susceptibility, and environmental factors in modulating these responses also warrants further investigation [27, 30, 31].

From a clinical perspective, there is a lack of prospective studies evaluating preventive strategies and treatment interventions for oral irAEs. Most current management approaches are extrapolated from therapies used in autoimmune diseases or other cancer treatment–related toxicities rather than being based on high-quality evidence specific to immunotherapy [6, 9]. In particular, the optimal use of topical vs systemic immunosuppressive agents and the long-term outcomes of these interventions remain unclear.

The impact of oral irAEs on oncologic outcomes is another area requiring further exploration. While severe systemic irAEs have been associated in some studies with improved antitumor responses, it is not well established whether oral toxicities carry similar prognostic implications or how their management may influence treatment efficacy [4].

Additionally, there is a need for the development of standardized dental care protocols tailored to patients receiving immunotherapy. Unlike chemotherapy and radiotherapy, for which well-defined oral care guidelines exist, recommendations for dental management in the context of ICIs are still evolving and are not uniformly implemented in clinical practice [10].

## Conclusion

Immunotherapy has introduced a distinct spectrum of oral adverse effects that are increasingly encountered in clinical practice. Although often underrecognized, these manifestations can significantly impair oral function, quality of life, and adherence to cancer treatment, while their overlap with other oral conditions complicates diagnosis.

This review presents a structured, dental-focused framework that integrates current evidence into a practical classification of oral findings, a clinically oriented diagnostic approach, and management considerations tailored to dental practitioners. By bridging oncologic knowledge with dental practice, it supports earlier recognition and more consistent management of oral irAEs.

Dental practitioners play a central and often underrecognized role in the identification, monitoring, and supportive management of these conditions. A proactive approach, including baseline assessment, regular follow-up, and close collaboration with oncology teams, is crucial to optimize patient outcomes and maintain continuity of cancer therapy.

However, current management strategies remain largely based on extrapolated evidence and expert consensus. Further prospective studies and the development of standardized clinical guidelines are necessary to strengthen the evidence base and refine dental care in this evolving field.
